# Philadelphia chromosome-negative myeloproliferative chronic neoplasms: is clonal hematopoiesis the main determinant of autoimmune and cardio-vascular manifestations?

**DOI:** 10.3389/fmed.2023.1254868

**Published:** 2023-10-17

**Authors:** Giovanni Fulvio, Chiara Baldini, Marta Mosca, Antonello di Paolo, Guido Bocci, Giuseppe Alberto Palumbo, Emma Cacciola, Paola Migliorini, Rossella Cacciola, Sara Galimberti

**Affiliations:** ^1^Department of Clinical and Experimental Medicine, Rheumatology, University of Pisa, Pisa, Italy; ^2^Department of Clinical and Translational Science, University of Pisa, Pisa, Italy; ^3^Department of Clinical and Experimental Medicine, Clinical Pharmacology, University of Pisa, Pisa, Italy; ^4^Department of Medical, Surgical Sciences and Advanced Technologies “G.F. Ingrassia” Hematology, University of Catania, Catania, Italy; ^5^Department of Medical, Surgical Sciences and Advanced Technologies “G.F. Ingrassia” Hemostasis, University of Catania, Catania, Italy; ^6^Department of Clinical and Experimental Medicine, Clinical Immunology, University of Pisa, Pisa, Italy; ^7^Department of Clinical and Experimental Medicine, Hemostasis, University of Catania, Catania, Italy; ^8^Department of Clinical and Experimental Medicine, Hematology, University of Pisa, Pisa, Italy

**Keywords:** CHIP, myeloproliferative neoplasms, autoimmune disease, cardiovascular disease, *JAK2*, *ROCK2*, *TET2*, *DNMT3A*

## Abstract

In this article, we reviewed the possible mechanisms linking the clonal hematopoiesis of indeterminate potential (CHIP) to chronic myeloproliferative neoplasms (MPNs), autoimmune diseases (ADs), and cardiovascular diseases (CADs). CHIP is characterized by the presence of clonal mutations with an allelic frequency >2% in the peripheral blood without dysplasia, overt hematological neoplasms, or abnormalities in blood cell count. The prevalence may reach 20% of elderly healthy individuals and is considered a risk factor for myelodysplastic neoplasms and acute leukemia. In MPNs, CHIP is often associated with mutations such as *JAK2V617F* or *DNMT3A, TET2*, or *ASXL1*, which exhibit a 12.1- and 1.7–2-fold increase in CADs. Specifically, *JAK2*-mutated cells produce excessive cytokines and reactive oxygen species, leading to proinflammatory modifications in the bone marrow microenvironment. Consequently, the likelihood of experiencing thrombosis is influenced by the variant allele frequency (VAF) of the *JAK2V617F* mutation, which also appears to be correlated with anti-endothelial cell antibodies that sustain thrombosis. However, *DNMT3A* mutations induce pro-inflammatory T-cell polarization and activate the inflammasome complex, while *TET2* downregulation leads to endothelial cell autophagy and inflammatory factor upregulation. As a result, in patients with *TET2* and *DNMT3A-*related CHIP, the inflammasome hyperactivation represents a potential cause of CADs. CHIP also occurs in patients with large and small vessel vasculitis, while ADs are more frequently associated with MPNs. In these diseases, monocytes and neutrophils play a key role in the formation of neutrophil extracellular trap (NET) as well as anti-endothelial cell antibodies, resulting in a final procoagulant effect. ADs, such as systemic lupus erythematosus, psoriasis, and arthritis, are also characterized by an overexpression of the Rho-associated coiled-coil containing protein kinase 2 (ROCK2), a serine/threonine kinase that can hyperactivate the JAK-STAT pathway. Interestingly, hyperactivation of *ROCK2* has also been observed in myeloid malignancies, where it promotes the growth and survival of leukemic cells. In summary, the presence of CHIP, with or without neoplasia, can be associated with autoimmune manifestations and thrombosis. In the presence of these manifestations, it is necessary to consider a “disease-modifying therapy” that may either reduce the clonal burden or inhibit the clonally activated JAK pathway.

## 1. Introduction

Philadelphia-negative chronic myeloproliferative neoplasms (MPNs) are clonal diseases originating from a single hematopoietic stem cell that causes excessive production of mature blood cells (1). According to the World Health Organization (WHO) and the International Consensus Classification (ICC) criteria, MPNs include polycythemia vera (PV), essential thrombocythemia (ET), and myelofibrosis (MF) (2, 3). The clonal origin of MPNs was identified in 1976 by studying X-chromosome inactivation patterns in the peripheral blood of female patients (4). Subsequently, the clonal nature of MPNs was further solidified by using multiple genetic analyses, such as whole-genome sequencing (WGS) and clonal mice model (5, 6). Currently, there is a lack of consensus regarding the appropriate nomenclature of mutations in MPNs; however, recently, the following classifications have been proposed: “*disease driver mutations*,” “*clonal driver mutations*,” “*passenger mutations*,” and “*variants of unknown significance*” (1).

The underlying cause of MPNs (1) is the acquisition of gain-of-function mutations in one of three “*disease driver genes*”: *Janus kinase 2* (*JAK2), Calreticulin (CALR*), and the *thrombopoietin receptor* (*MPL)*. Clearly, these mutations alone are sufficient to initiate and promote MPN without the need for additional cooperating mutations (6, 7). The *JAK2-V617F* mutation is present in most of the PV patients and in approximately half of the patients with ET or MF (8) that can show *CALR* or *MPL* mutations or be “triple negative” (9–11). Regardless of the presence or absence of specific driver gene mutations, the constitutive activation of the Janus kinase-signal transducer and activator of the transcription (JAK/STAT) signaling pathway is a common feature in all MPNs (12). “*Clonal driver mutations*,” when present alone, do not induce a specific MPN phenotype; anyway, they can modify the features of the disease when combined with one of other “disease driver” mutations that can be found across a range of myeloid malignancies (13–18). Most of these mutations involve epigenetic regulator genes (*TET2, ASXL1, DNMT3A, EZH2, and IDH1/2*), genes of messenger RNA splicing (*U2AF1, SF3B1, SRSF2, and ZRSR2*), or DNA repair (*PPM1D and TP53*), with *TET2, ASXL1, and DNMT3A* accounting for up to 80% of all cases of clonal hematopoiesis (19). “*Passenger mutations*” refer to genetic alterations that do not contribute to disease pathogenesis and manifestations. In some instances, predicting whether a gene mutation will alter function can be challenging, particularly since many mutations have not been extensively studied. Consequently, these mutations are initially categorized as “*variants of unknown significance,”* but over time, as our understanding of these mutations grows, they may be reclassified as disease driver mutations or passenger mutations (1).

The molecular mechanisms of clonal hematopoiesis underlying hematological manifestations were extensively explored and described. Nevertheless, the prognosis and the treatment were mainly associated with non-hematological manifestations: inflammation/autoimmunity and subsequent thrombosis.

Recently, the molecular pathogenesis of non-hematological manifestations has been recognized as increasingly important after the discovery of the clonal hematopoiesis of indeterminate potential (CHIP). CHIP is characterized by the presence of clonal mutation with an allelic frequency >2% in peripheral blood, without dysplasia, overt hematological neoplasms, or abnormalities in blood cell count, which represents a risk factor for myelodysplasia and leukemia (19–21). It is worth noting that mutations can lead to autoimmunity and thrombosis even in the absence of neoplasia. In fact, the prevalence of CHIP is significantly higher in individuals with autoimmune diseases (ADs) when compared to the normal population, being the highest in large vessel vasculitis and small vessel vasculitis, with rates of 33.3 and 30.4%, respectively (as shown in Table 1) (22–27). Meanwhile, patients with CHIP, represented by *JAK2V617F* mutation, had a 12.1-fold increase in cardiovascular diseases (CADs), while CHIP sustained by *DNMT3A, TET2*, or *ASXL1* had a 1.7–2-fold increase in CADs (28). These associations taken together suggest that clonal hematopoiesis is associated with autoimmunity and inflammation independently from the presence of neoplasia.

**Table 1:** Prevalence of overall and single somatic mutations in autoimmune diseases.

**References**	**ADs**	**CHIP overall prevalence n (%)**	**CHIP mutation**	**Single CHIP prevalence n (%)**
Savola et al. (22)	Rheumatoid arthritis	10/59 (16.9)	DNMT3A TET2 GNAS CBL TP53 ASXL1	4/59 (6, 8) 3/59 (5, 1) 2/59 (3, 4) 1/59 (1, 7) 1/59 (1, 7) 1/59 (1, 7)
Ricard et al. (23)	Systemic Sclerosis	13/90 (14, 4)	DNMT3A ATM SETBP1 NF1 SF3B1 TP53 TET2 CBL	7/90 (7, 8) 2/90 (2, 2) 1/90 (1, 1) 1/90 (1, 1) 1/90 (1, 1) 1/90 (1, 1) 1/90 (1, 1) 1/90 (1, 1)
Arends et al. (24)	Anti-neutrophil cytoplasmic antibody (ANCA)-associated vasculitides	34/112 (30, 4)	DNMT3A TET2 ASXL1 SRSF2 TP53 PPM1D RAD21 BCCOR ETV6 GNAS GNB1 KDM6A SF3B1	19/112 (17, 0) 7/112 (6, 3) 5/112 (4, 5) 3/112 (2, 7) 2/112 (1, 8) 2/112 (1, 8) 2/112 (1, 8) 1/112 (1, 8) 1/112 (1, 8) 1/112 (1, 8) 1/112 (1, 8) 1/112 (1, 8) 1/112 (1, 8)
David et al. (25)	Systemic lupus erythematosus	47/438 (10, 7)	DNMT3A TET2 GNAS ASXL1 SH2B3 TP53 CBL ETV6 JAK2 KDM6A NFE2 PPM1D SETBP1 SMC3	39/438 (8, 9) 7/438 (1, 6) 3/438 (0, 7) 2/438 (0, 5) 2/438 (0, 5) 2/438 (0, 5) 1/438 (0, 2) 1/438 (0, 2) 1/438 (0, 2) 1/438 (0, 2) 1/438 (0, 2) 1/438 (0, 2) 1/438 (0, 2) 1/43 8 (0, 2)
Park et al. (26)	Behçet's disease	13/117 (11, 1)	DNMT3A TET2 ASXL1 STAG2 IDH2	9/117 (7, 7) 2/117 (1, 7) / / /
Papo et al. (27)	Giant cell arteritis	5/15 (33, 3)	DNMT3A CBL	4/15 (26, 7) 1/15 (6, 7)

Some patients carried more than one mutation. Ads, autoimmune diseases; CHIP, Clonal hematopoiesis of indeterminate potential.

The clinical impact of CHIP is quite evident. In addition to being associated with ADs, it has been reported that CHIP increases the risk of death in individuals with hematological neoplasms by 40%. However, only 0.5% of them actually succumb to cancer. Additionally, MPNs have a higher incidence of CADs, including ischemic stroke (HR 2.6) and premature myocardial infarction (HR 4.0), which are not related to traditional cardiovascular risk factors (19, 29).

This narrative review aims to investigate the association between clonal hematopoiesis and autoimmune and cardiovascular manifestations. In particular, we have described how the immune system can modify clonal hematopoiesis progression and how the molecular pathogenesis of clonal hematopoiesis might be related to ADs and CADs. The search was conducted across various electronic databases (PUBMED, MEDLINE) to identify relevant articles published in the English language using the following keywords: “myeloproliferative disease,” “autoimmune disease,” “autoimmunity,” “thrombosis,” “clonal haematopoiesis,” and “inflammation.” No geographic restrictions were imposed, ensuring a global representation of research findings. We included all articles relevant to the abovementioned purposes, while excluding the others.

## 2. Clonal hematopoiesis of driver mutations in myeloproliferative chronic neoplasms

*JAK2* plays a crucial role as a signal transducer in the hematopoietic stem cell (HSC) compartment, facilitating growth, and differentiation in response to cytokine receptors without intrinsic kinase activity. These receptors include the erythropoietin receptor, the thrombopoietin receptor, and the granulocyte colony-stimulating factor receptor. The presence of *JAK2V617F* mutation leads to the activation of *STAT* with subsequent increased levels of red blood cells, neutrophils, and platelets (30–33). In fact, there is a direct correlation between the allele burden of *JAK2V617F* and various measurements of stimulated erythropoiesis and myelopoiesis (34).

In addition to JAK-STAT signaling, other pathways, such as phosphatidylinositol-3-kinase/AKT (PI3K/AKT) and the RAS/mitogen-activated protein kinases (RAS/MAPK), are also overactivated by the *JAK2V617F* mutation (35). Specifically, *JAK2*-mutated HSCs produce excessive cytokines and reactive oxygen species (ROS), leading to inflammatory modifications in the bone marrow microenvironment. The inflammatory environment further strengthens the competitive advantage of the clonal cells and contributes to their genomic instability (36, 37).

Moreover, the excessive cellularity in the bone marrow induces a state of pseudohypoxia activating hypoxia-inducible factor (HIF). High levels of HIF, in turn, enhance HSC self-renewal. This effect is achieved through the downregulation of LKB1/STK11, a protein known to negatively regulate HSC self-renewal (38).

*CALR* mutations in MPNs involve insertions and/or deletions in exon 9, which cause a frameshift and lead to the production of a new positively charged C-terminus in the mutant CALR protein. This unique C-terminus in the mutant *CALR* enables its binding to *MPL* (myeloproliferative leukemia) protein, initiating activation of the *MPL/JAK/STAT* signaling pathway (39).

The STAT family is also a key component in the pathogenesis of MPNs. In fact, deletion of *STAT5* in transgenic mouse models expressing *JAK2V617F* mutation abolishes the disease phenotypes. Moreover, *STAT1* has been identified as a promoter of megakaryopoiesis downstream of *GATA1*. In a mouse model of MPN, deletion of *STAT1* in the presence of *JAK2V617F* leads to a reduction in megakaryopoiesis and a preference for erythropoiesis. Conversely, loss of *STAT3* in hematopoietic cells enhances *JAK2V617F*-driven thrombopoiesis, potentially mediated by increased expression and activation of *STAT1*. In fact, in MPNs, *STAT1*, and *STAT3* activation are reciprocally regulated, and imbalances in their activity can change cytokine and growth factor signals (40, 41).

However, MPNs are also characterized by disease driver mutations: 27% of patients carry *TET2* mutations and 16% carry *DNMT3A* mutations, which are the most frequent gene aberrations sustaining CHIP (42). Clearly, it has been reported that *DNMT3A* mutations activate the inflammasome complex and induce proinflammatory T-cell polarization, which is responsible for cardiovascular effects, as demonstrated in the murine model, where *DNMT3A* deletion in stem cells induces cardiac hypertrophy and reduces cardiac function (43). Analogously, in another murine model, *TET2* downregulation leads to endothelial cell autophagy and inflammatory factors upregulation (44). It has been reported that 5.1% of subjects with atherosclerosis have CHIP, and the presence of *TET2*-related clonal hematopoiesis significantly increases the risk of adverse outcomes (45). Moreover, in a series of 125 MPN patients, 28% had thrombotic events; similarly, in ET patients, the *TET2* mutations were an independent risk factor for thrombosis (HR 4.1). However, they did not have an effect on CADs in patients affected by PV (46).

**Figure 1:**
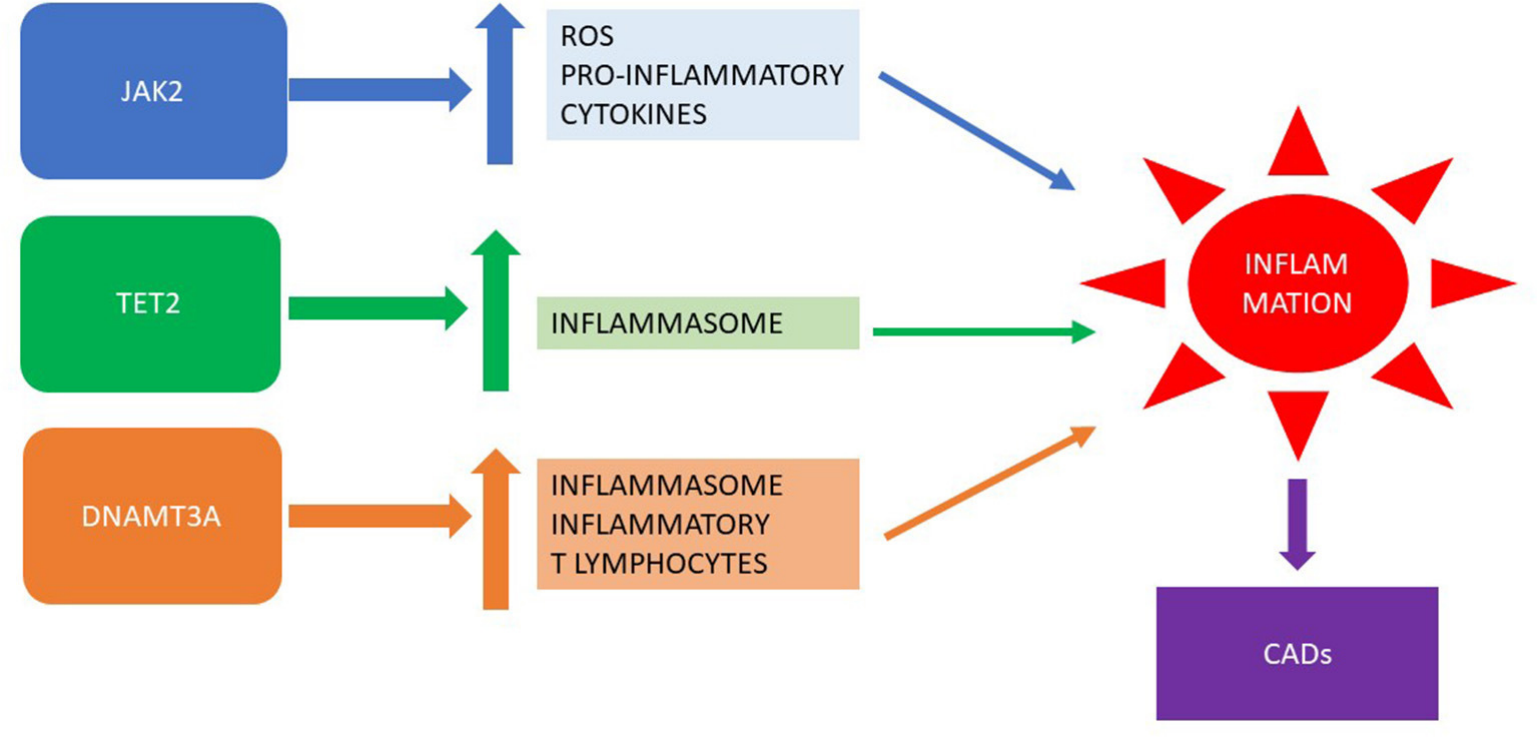
Clonal hematopoiesis of driver mutations in MPNs: inflammation and CADs. MPN, myeloproliferative neoplasm; CADs, cardiovascular diseases.

The relationship between clonal hematopoiesis of driver mutations in MPNs and CADs is depicted in Figure 1.

## 3. Risk of thrombosis in MPNs

A meta-analysis revealed that, upon initial diagnosis of MPN, thrombosis was observed in approximately 20% of patients: arterial thrombosis was prevalent in approximately 16.2% of subjects, while venous thrombosis accounted for 6.2% of cases. Common thrombotic events included cerebrovascular disease or transient ischemic attack, coronary heart disease, and deep venous thrombosis (47). In the MPNs, thrombosis resulted from the interactions between inflammatory and procoagulant factors and microparticles, along with qualitative and quantitative abnormalities of red cells, leukocytes, platelets, and endothelial cells.

An increased number of red blood cells can lead to the development of venous or arterial thrombosis by an augmented blood viscosity; however, this factor alone is not sufficient to explain the heightened thrombotic tendency observed in PV. This can be evidenced by the fact that individuals with comparable or even higher levels of secondary erythrocytoses, such as those caused by high altitude or chronic obstructive lung disease, generally do not exhibit increased susceptibility to thrombosis (48). Nevertheless, it is worth noting that sustained normalization of hematocrit levels (to below 45%) in PV patients is associated with a reduction in thrombotic events, indicating that erythrocytosis plays a significant role as a risk factor for thrombosis in PV (49).

There are no confirmed associations between elevated platelet levels and the occurrence of initial thrombotic and vascular complications in patients. Surprisingly, individuals with essential thrombocythemia and extreme thrombocytosis tend to experience bleeding complications rather than thrombotic events, which may be due to acquired von Willebrand disease (48, 50, 51).

The association between leucocytosis and thrombosis risk in patients with MPNs is still not fully established but demonstrates some evidence. The relationship between leucocytosis and thrombosis was primarily observed in arterial thrombotic events and predominantly in patients with ET. Studies have linked leukocyte counts above 15.3 × 10^9^/L in PV and 11.3 × 10^9^/L in ET with an increased risk of thrombosis (52, 53). A meta-analysis further confirmed that leucocytosis is an established risk factor for thrombosis, and it is likely to be independent of other factors (54).

## 4. Influence of the immune system on clonal hematopoiesis developments

ADs and inflammation may play an important role in inducing or avoiding clonal hematopoiesis development. Indeed, the prevalence of *JAK2V617F* clonal CHIP is significantly higher than the *CALR-*mutated one, resulting in a higher frequency of MPNs with *JAK2* mutation when compared to *CALR* mutation. The observed differences in prevalence may be influenced by the ability of HSCs carrying *driver disease mutations* to escape immune surveillance. It has been noted that certain healthy individuals possess memory T-cells that recognize the mutated CALR neoantigen; however, they do not exhibit CALR-mutated CHIP. This suggests that the lower prevalence of *CALR*-mutated CHIP could be attributed to a more efficient elimination of *CALR*-mutated cells at the pre-CHIP stage by the immune system, likely in a HLA-restricted manner (55). Indeed, it was demonstrated that several peptides capable of binding numerous MHC class I molecules originate from *CALR* and *MPL* mutations (56). However, in *JAK2V617F* mutation, peptides have only weak interactions with any HLA allotype, reducing their recognition by the immune system. Moreover, the activation of JAK/STAT signaling by *JAK2V617F* leads to increased expression of programmed death ligand 1 (PD-L1). This combination of factors allows cells expressing JAK2 mutation to evade immune surveillance (57).

**Figure 2:**
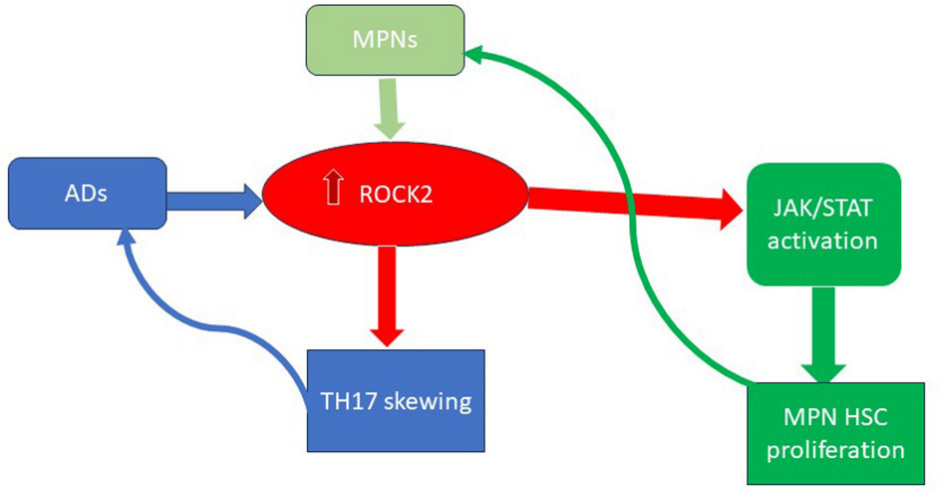
The key role of ROCK2 activation in ADs and MPNs. ROCK2, Rho-associated coiled-coil containing protein kinase 2; MPN, myeloproliferative neoplasms; Ads, autoimmune diseases; HSC, hematopoietic stem cell.

Additionally, uncontrolled inflammation could also promote CHIP or MPN development. The presence of IL-1β, an inflammatory cytokine, significantly promotes early clonal expansion in MPNs (58). Autoimmune diseases, such as systemic lupus erythematosus (SLE), psoriasis, and arthritis, are also characterized by an overexpression of the Rho-associated coiled-coil containing protein kinase 2 (ROCK2). ROCK2 is a cytoplasmic serine/threonine kinase that can hyperactivate the JAK-STAT pathway. Interestingly, hyper-activation of *ROCK2* has also been observed in myeloid malignancies, where it promotes the growth and survival of leukemic cells through the constitutive activation of the PI3K/Rho/ROCK/myosin light chain pathway (59, 60). Moreover, during the immune response, ROCK2 controls the TCR signaling and, through IL21 and IL17, sustains the TH17 generation, thus being implicated in the autoimmunity onset (see Figure 2) (61).

## 5. Clonal hematopoiesis causes thrombosis through immune dysregulation

**Figure 3:**
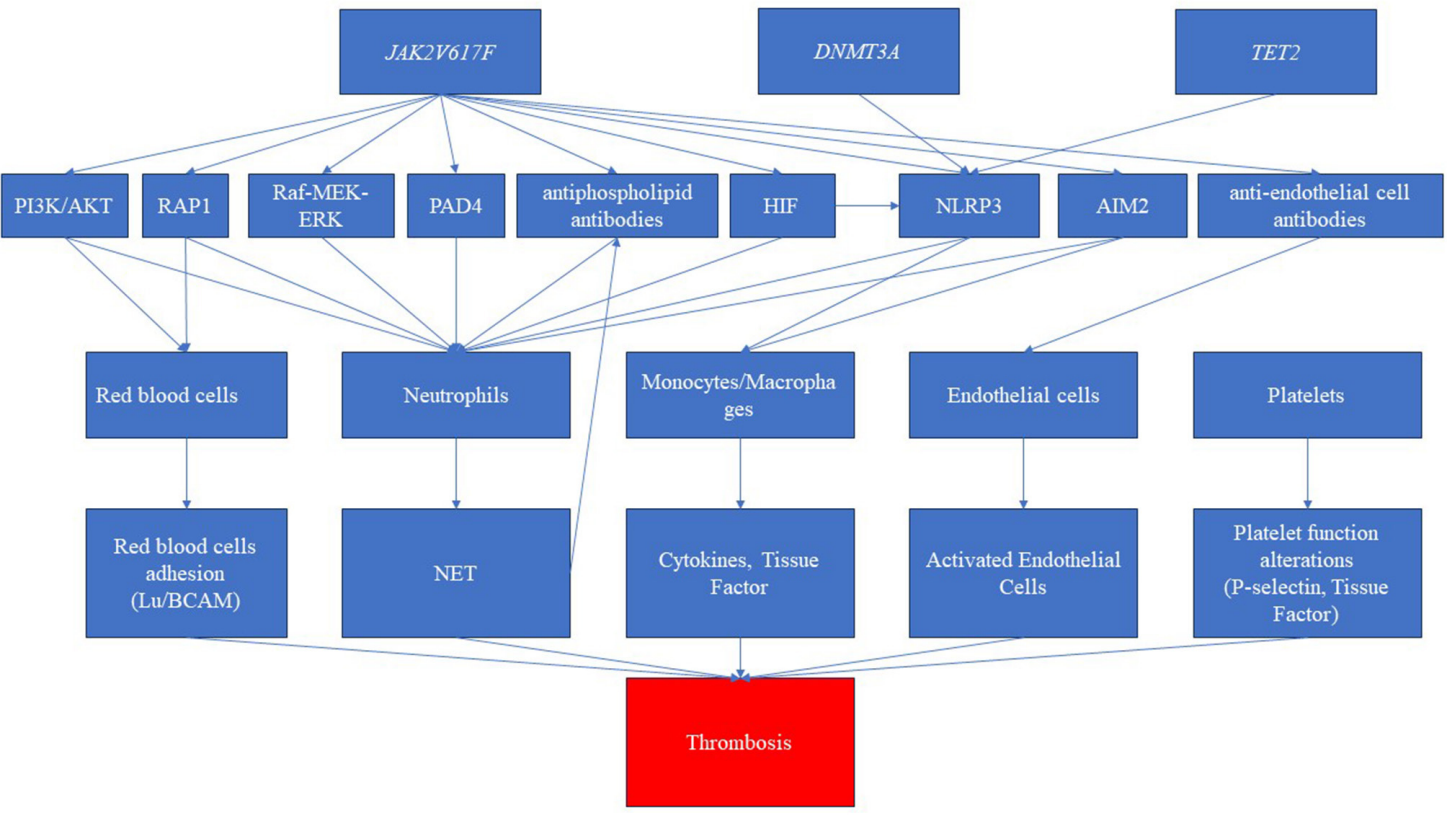
Pathways related to clonal hematopoiesis involved in immune dysregulation and thrombosis. The disease driver mutation of Janus kinase (JAK2V617F) represents a pivotal trigger, inducing a cascade of intricate molecular pathways. These pathways encompass the activation of diverse routes such as phosphatidylinositol-3-kinase/AKT (PI3K/AKT), RAS-related protein 1 (RAP1), rapidly accelerated fibrosarcoma (Raf)/mitogen-activated protein kinase (MEK)/extracellular signal-regulated kinase (ERK), and hypoxia-inducible factor (HIF). Furthermore, this mutation elicits the activation of inflammasomes, notably NLR family pyrin domain containing 3 (NLRP3) and absent in melanoma 2 (AIM2), amplifying the inflammatory milieu. Simultaneously, the JAK mutation is associated with the production of autoantibodies, specifically antiphospholipid antibodies and anti-endothelial antibodies. Consequently, these intricate molecular perturbations culminate in the dysregulation of crucial blood cell populations. While red blood cells, platelets, and endothelial cells shift toward a procoagulant phenotype, neutrophils and monocytes produce neutrophil extracellular traps (NETs) and cytokines, respectively. This intricate interplay culminates in a pronounced prothrombotic state. Furthermore, the clonal driver mutations, particularly DNMT3 and TET2, have been shown to possess the capability to activate inflammasome pathways, further intensifying the prothrombotic environment.

Clonal hematopoiesis, autoimmune diseases, and thrombotic manifestations are strictly linked to each other (17) (see Figure 3). However, the links among them are not well defined. The *JAK2V617F* mutation is correlated with a heightened risk of thrombosis, whereas *CALR* mutations are associated with a lower risk compared to *JAK2* and *MPL* mutations (62, 63). Furthermore, in MPNs, the likelihood of experiencing thrombosis is influenced by the variant allele frequency (VAF) of *JAK2V617F* (64). Notably, patients with PV exhibited a higher incidence of thrombotic events. This difference may be attributed to the varying associations between specific driver gene mutations and thrombosis risk factors. Moreover, in a large population-based study, individuals with a personal history of autoimmunity exhibited a 20% higher risk of developing MPN, particularly in cases associated with vasculitis (65). The presence of an autoimmune disease remained an independent prognostic factor for thrombosis, even when considering the cardiovascular risk (HR 5.42) (66). This may suggest that specific mutations may be associated with alteration of the clonal cells (red blood cells, platelets, white blood cells, and endothelial cells) and the surrounding non-clonal environment, ultimately leading to thrombosis formation. First, for example, *JAK2V617F* mutation in red blood cells triggers signaling cascades that lead to the activation of RAS-related protein 1 (*RAP1)* and *AKT*, ultimately promoting the adhesion of red blood cells mediated by Lu/BCAM (67).

In MPNs, different platelet function alterations have been observed, particularly in individuals with *JAK2*-mutated ET or a history of thrombosis (68). These abnormalities include increased levels of P-selectin and platelets positive for tissue factor (TF), some being specific to MPNs. These abnormalities are likely inherent to platelets derived from mutant stem cells; in fact, there is a correlation between the burden of *JAK2* mutation and platelet activation (69). However, it is important to note that none of these platelet defects has been proven to be causative for clinical thrombosis. In fact, in MPNs, testing conditions can dramatically modify *in vitro* platelet function; conversely, analytical conditions closer to the physiological revealed normal/supranormal function (70).

The association between white blood cells and thrombosis highlights the primary role of leucocyte dysfunction in the genesis of thrombosis, mainly through neutrophils and monocytes. *JAK2V617F* expression is associated with neutrophil activation, in particular, with increased neutrophil extracellular trap (NET) formation, one of the major causes of cancer-associated thrombosis (71). *JAK*, through its activated pathways and regulatory molecules, such as STAT, AKT, and Raf-MEK-ERK, may regulate the NET formation both in normal neutrophils and neutrophils in pathological situations. In addition, in JAK2-mutated PV patients, peptidyl arginine deiminase (PAD4), HIF-mediated gene expression, and RAP1 were increased, leading to the production of NET (72–74). Moreover, *RAP1* activates integrin signaling and enhances the adhesion of clonal neutrophils in PV. Interestingly, it was demonstrated that the inhibition of JAK2 signaling may reduce thrombosis in MPNs by the inhibition of NET formation (75, 76). As regards monocytes, *JAK2*-mutated macrophages showed increased expression of proinflammatory cytokines and chemokines, i.e., IL-1β, IL-6, iNOS, TNF-α, and MCP-1 (77, 78). The main contributors to cytokine production in monocytes are the inflammasome, as NLR Family Pyrin Domain Containing 3 (NLRP3) and Absent in Melanoma 2 (AIM2). AIM2 is a cytoplasmic sensor and is activated by dsDNA from bacterial, viral, or cellular origin (79). *JAK2V617F* mutation leads to metabolic and proliferative changes in macrophages resulting in replication stress. The presence of damaged oxidative DNA in hyperproliferative cells results in *AIM2* activation. Simultaneously, an excess of HIF alters iron metabolism increasing cellular labile iron, thus inducing ROS, the activator of *NLRP3* (80–82). The activation of both the inflammasomes leads to the massive production of IL-1β that ulteriorly promotes the NET formation and consequently thrombosis (83). Interestingly, the CANTOS trial demonstrated that canakinumab, a therapeutic monoclonal antibody targeting interleukin-1β, reduces recurrent cardiovascular events. The inflammasome, moreover, causes pyroptosis, a proinflammatory programmed cell death mediated by Gasdermin D. Inflammasome-mediated pyroptosis releases tissue factor from macrophages and activates coagulation (84, 85).

Focusing on vessels, endothelial cells (ECs) carrying the *JAK2V617F* mutation have been detected in patients with MPNs. Mesodermal pluripotent stem cells, indeed, have the capacity to differentiate into both hematopoietic and endothelial lineages (86, 87). In instances where vascular endothelial integrity is compromised, re-endothelialization can occur through the outgrowth of neighboring intact endothelium or by repopulating EC progenitors. Consequently, a somatic MPN driver mutation can be implanted in endothelium (88, 89) and the injury or activation of ECs leads to a shift in their properties, from anti- to pro-thrombotic. *In vivo*, activation of ECs in MPNs is reflected by elevated biomarkers like the von Willebrand factor (90).

The immune dysregulation in MPNs may involve adaptive immunity, in particular, autoantibody production, as demonstrated by a very high frequency of organ/non-organ specific antibodies (57%), anti-erythrocyte antibodies (45%), and anti-platelet antibodies (15%) (91). In PV patients, the *JAK2V617F* allele burden was correlated with anti-endothelial cell antibodies (AECAs) that are capable of recognizing activated endothelial cells resulting in thrombosis (92). Interestingly, a similar mechanism has been described in small vessel vasculitis (93).

There is a notable connection between *JAK2V617F*-mutated MPNs and definite antiphospholipid syndrome (APS), an autoimmune thrombotic syndrome caused by antiphospholipid antibodies (aPL). Both APS and MPNs exhibit significant upregulation of proinflammatory cytokines and an enhanced formation of NETs (94). In APS, the release of NETs is associated with the levels of antiphospholipid antibodies (aPL), and aPL can induce NETosis in neutrophils from healthy individuals (95). Moreover, aPL are also directed toward mitochondria (96). Notably, in PMF, a higher prevalence of mitochondrial DNA (mtDNA) and anti-mitochondrial DNA (anti-mtDNA) was found (82). Furthermore, In SLE, it was demonstrated that anti-mitochondrial antibodies are associated with NET (97, 98). Overall, these findings highlight the strong interplay between *JAK2V617F*-mutated MPNs, APS, NET formation, and the dysregulation of proinflammatory cytokines, providing insights into the complex and interconnected nature of these conditions.

Finally, other mutations also can play a role in thrombosis pathogenesis. In patients with *TET2* and *DNMT3A* clonal hematopoiesis, an inflammasome hyperactivation in myeloid cells was noticed, which represents the potential cause of cardiovascular disease (99, 100). TET2 mutation is not a standalone risk factor for thrombosis. The order of acquisition, whether it occurs first or after other mutations, is crucial. When *TET2* mutation is acquired before *JAK2* mutation, the thrombotic risk is higher (101).

## 6. Conclusion

The immune system has a primary role in clonal hematopoiesis development. Impaired immune surveillance and high inflammation (for example, by IL-1β and ROCK2 pathway) may facilitate CHIP occurrence. However, clonal hematopoiesis may directly alter the immune system, in particular, the neutrophils (NET formation), the macrophages (inflammasome and release of tissue factor), and the autoantibody production (anti-endothelial cell antibodies and antiphospholipid antibodies), leading to thrombosis. Interestingly, among the autoimmune diseases, CHIP is more prevalent in large vessel vasculitis and small vessel vasculitis, in which it is known to play the key role of, respectively, monocyte and neutrophil/NET formation (102–105). Furthermore, the most commonly associated autoimmune diseases with MPNs are vasculitis (65). With regard to autoantibodies, anti-endothelial cell antibodies are frequently detected in different autoimmune diseases, exhibiting a procoagulant effect by activating endothelial cells (106), as proven by the complex thrombotic action exerted by the antiphospholipid antibodies. Indeed, NET formation results in antibodies against mitochondria, including anti-phospholipid antibodies. These antibodies are frequently detected in systemic lupus erythematosus, and their pathogenetic association with thrombosis is clearly evident.

In summary, the presence of clonal hematopoiesis, with or without neoplasia, can be associated with autoimmune manifestations and thrombosis. In the presence of these manifestations, it seems necessary to consider a “disease-modifying therapy” that may reduce the clonal burden or inhibit the clonally activated JAK pathway. Recently, it has been shown that interferon may reduce clonal burden, while ruxolitinib, a JAK1/2 inhibitor, may inhibit the JAK-STAT pathway (107). Ultimately, the comprehension of the molecular pathogenesis of autoimmune and thrombotic manifestations provoked by clonal hematopoiesis might allow us a targeted therapy toward the era of precision medicine.

## Author contributions

GF: Writing—original draft. CB: Writing—original draft. MM: Writing—review and editing. AP: Writing—review and editing. GB: Writing—review and editing. GP: Writing—review and editing. EC: Writing—review and editing. RC: Writing—review and editing. PM: Writing—review and editing. SG: Writing—review and editing.
